# AARS and CACNA1A mutations: diagnostic insights into a case report of uncommon epileptic encephalopathy phenotypes in two siblings

**DOI:** 10.3389/fneur.2024.1376643

**Published:** 2024-04-15

**Authors:** Vanessa I. Romero, Samantha Sáenz, Benjamín Arias-Almeida, Daniela DiCapua, Kazuyoshi Hosomichi

**Affiliations:** ^1^School of Medicine, Universidad San Francisco de Quito, Quito, Ecuador; ^2^Neurology Service, Hospital de Especialidades Eugenio Espejo, Quito, Ecuador; ^3^Laboratory of Computational Genomics, Tokyo University of Pharmacy and Life Sciences, Tokyo, Japan

**Keywords:** AARS, CACNA1A, case review, epilepsy, progressive cognitive decline, epileptic encephalopathy

## Abstract

Epilepsy, characterized by recurrent seizures, impacts 70–80% of patients, leading to cognitive deficits. The intricate relationship between seizure control and cognitive impairment remains complex. Epileptic encephalopathy (EE), an intensified form often rooted in genetic factors, is detectable through next-generation sequencing, aiding in precise diagnoses, family counseling, and potential treatment strategies. We present a case involving two sisters with refractory generalized seizures evolving into dysarthria, dysphagia, ataxia, and cognitive decline. Despite normal physical exams, abnormal electroencephalogram results consistent with epilepsy were noted. Whole Exome Sequencing identified heterozygous variants in the alanyl-tRNA synthetase (AARS) and Calcium Voltage-Gated Channel Subunit Alpha 1 (CACNA1A) genes. The AARS variant (c.C2083T, p.R695*) was maternal, while the CACNA1A variant (c.G7400C, p.R2467P) was paternal. Patients A and B exhibited a unique blend of neurological and psychiatric conditions, distinct from common disorders that begin adolescence, like Juvenile Myoclonic Epilepsy. Whole Exome Sequencing uncovered an AARS gene and CACNA1A gene, linked to various autosomal dominant phenotypes. Presence in both parents, coupled with familial reports of migraines and seizures, provides insight into accelerated symptom progression. This study underscores the importance of genetic testing in decoding complex phenotypes and emphasizes the value of documenting family history for anticipating related symptoms and future health risks.

## Introduction

Epilepsy, characterized by recurrent seizures, is a prevalent neurological disorder that induces irreversible brain damage through frequent and recurrent seizures, leading to cognitive changes (1, 2). Cognitive impairment, observed in 70–80% of epilepsy patients, manifests as memory loss, cognitive slowing, and attention deficits, significantly impacting daily functions (3). Multiple factors contribute to cognitive dysfunction, including organic brain lesions, uncontrolled seizures, drug treatment, and individual mental abilities (2, 4). Conflicting findings exist regarding the relationship between seizure control and cognitive impairment, emphasizing the complex interplay between epilepsy and cognitive function across diverse populations and contexts (1, 5).

Epileptic encephalopathy (EE) represents a severe form of epilepsy in which epileptic activity exacerbates cognitive and behavioral impairments beyond the anticipated effects of the underlying pathology alone (6). Manifesting across various age groups, EE is commonly associated with genetic etiologies, often identified through next-generation sequencing (NGS) techniques (7). One significant genetic aspect involves disruptions in voltage-gated channels, particularly voltage-gated sodium channels (VGSCs) such as SCN1A, SCN2A, and SCN8A, implicated in epilepsies of varying severity (8–10). Potassium channelopathies, including KCNQ2 and KCNQ3 mutations, and disruptions in inwardly rectifying potassium (Kir) channels (e.g., KCNB1) also contribute to EE, affecting the frequency and duration of action potentials (11, 12). Calcium channelopathies, particularly mutations in CACNA1A, have been linked to various neurological phenotypes, including epilepsy, hemiplegic migraine, and episodic ataxia (13–17). Understanding the intricate relationship between genetic factors and channelopathies in epileptic encephalopathy is crucial for precise diagnosis, management, and genetic counseling.

Our patients presented with unexplained neurological signs and symptoms, for which we performed next-generation sequencing. Genetic testing, such as Whole Exome Sequencing (WES), is increasingly utilized in different specialties, including neurological and neurogenic conditions, particularly in phenotypes with broad variability and overlap of potential genetic causes (18). WES facilitates accurate diagnosis for these patients, enabling family counseling, recurrence prevention, and treatment, when available (19). Additionally, next-generation sequencing allows the association of novel phenotypes with previously described genes, as observed in our patients (18).

## Patients’ description

**Figure 1:**
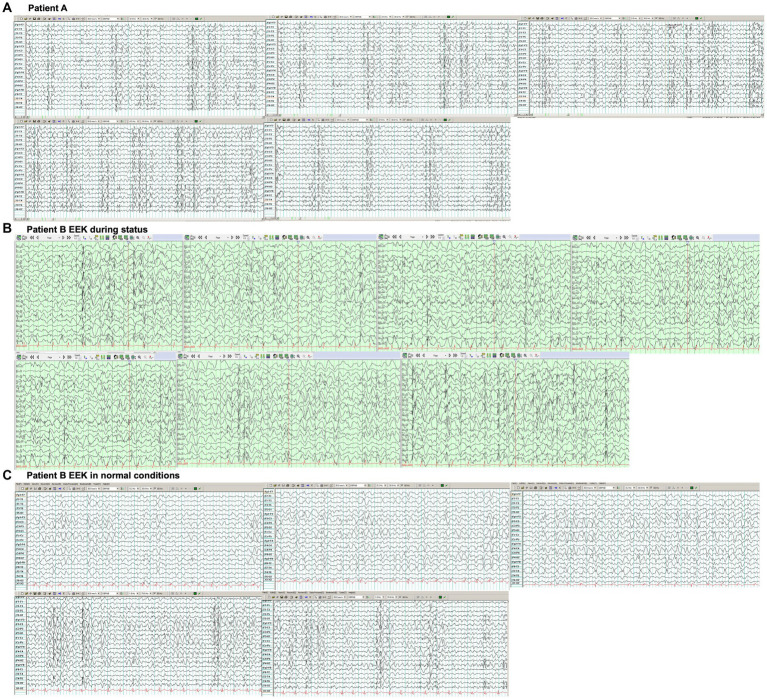
**(A)** Electroencephalogram of patient A with a bipolar montage showed generalized slowness in the background activity, along with abundant multifocal epileptic activity and generalized activity. **(B)** Electroencephalogram of patient B. Electroencephalogram showing reported generalized slowness of background activity with abundant multifocal epileptic activity, predominantly affecting the posterior regions, consistent with a non-convulsive status epilepticus. **(C)** Electroencephalogram in normal conditions showed generalized slowness in the background activity, along with abundant multifocal epileptic activity and generalized activity.

Patient A, a 20-year-old Hispanic female, was previously in good health. At the age of 15, she experienced the onset of generalized myoclonic-tonic–clonic seizures (HP: 0032795) that proved refractory to medical treatment. One year after the onset of epilepsy, she began to manifest symptoms of dysarthria (HP: 0001260), dysphagia (HP: 0002015), and ataxia (HP: 0001251). By the age of 18, she exhibited a progressive cognitive decline (HP: 0001268), losing the ability to speak, walk, and eat. During the physical examination, no dysmorphic features were observed. Her language skills were characterized by mutism (HP: 0002300). She demonstrated alexithymia (HP: 0031433), with delayed spoken language comprehension (HP: 5200240). Muscular strength was assessed at 4/5 in all four extremities using the Medical Research Council (MRC) Scale, accompanied by areflexia (HP: 0001284), erratic myoclonus (HP: 0025357), and gait ataxia (HP: 0002066). The electroencephalogram (Figure 1) revealed generalized slowness in the background activity, along with abundant multifocal epileptic activity and generalized activity. Cerebral magnetic resonance image did not show structural lesion. At the time of our visit, the patient was highly dependent and required assistance with her daily living activities like bathing, dressing, toileting, transferring, continence and feeding (KATZ ADL of 0/6).

Patient B, the sister of Patient A, is a 17-year-old Hispanic female who was also previously in good health. She presented to the hospital with a one-year history of generalized myoclonic-tonic–clonic seizures primarily occurring during sleep, persisting daily despite pharmacological treatment. Alongside epilepsy, she exhibited psychiatric symptoms characterized by delusional perception (HP: 5200424), auditory hallucination (HP: 0008765), emotional lability (HP: 0000712), and insomnia (HP: 0100785), coupled with progressive cognitive decline that led to her abrupt discontinuation of school attendance. During the physical examination, no dysmorphic features were observed, but she displayed confusion (HP: 0001289), bradyphrenia (HP: 0031843), and delayed spoken language comprehension (HP: 5200240). Also, gait ataxia (HP: 0002066) was observed. Magnetic resonance imaging of the brain revealed no abnormalities. The electroencephalogram (Figure 1) reported generalized slowness of background activity with abundant multifocal epileptic activity, predominantly affecting the posterior regions, consistent with a non-convulsive status epilepticus (Supplementary Figure 1). At the time of our visit, the patient was dependent and required assistance with her daily living activities like bathing, dressing, transferring, continence and feeding (KATZ ADL of 1/6).

**Figure 2:**
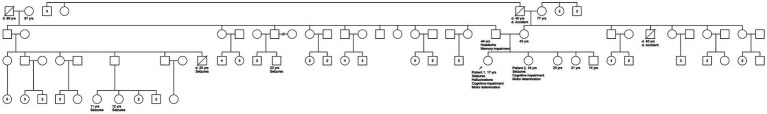
Family pedigree. The father had migraine and memory impairment during adolescence. Relatives from the father side have seizures.

Chromosome microarray analysis for Patient B confirmed the absence of copy number changes or copy-neutral regions. The patient’s father, who reported a history of headaches and memory impairment (HP: 0002354) during adolescence, has since resolved those symptoms and has not experienced any seizures. The patient’s mother is in overall good health. Additionally, two female and two male cousins from the patient’s father’s side have presented with seizures but without any other accompanying neurological symptoms (Figure 2). We conducted a Whole Exome Sequencing (WES) analysis on the peripheral blood samples obtained from both the sisters and their parents to ascertain the genetic condition. The parents were not consanguineous but were born and live in the same rural area.

We used a capture of target regions using probes, followed by next-generation sequencing with Illumina technology. The total number of reads was 41,660,733, whereas that of the mapped reads was 41,603,016 (99.86%). We aligned the raw data using the Burrows–Wheeler Aligner software, sorted and merged the data using the Picard tools software, and identified the nucleotide variants (SNV) and insertions or deletions (indel) using GATK. The GRCh37 version of the human genome was taken as reference.

After performing WES, we identified 1,529 variants causing non-synonymous, stopgain, and frameshift changes; selected those with minor allele frequency (<0.01) in various databases; and detected two variants. The first one a nonsense heterozygote C > T (c.C2083T) variant of uncertain significance (db SNP rs761043713) in 15th exon of alanyl-tRNA synthetase (AARS), resulting in a arginine to a premature stop codon change at position 695 (p.R695*). The nonsense variant has a frequency of 0.0000323 in The Genome Aggregation Database (gnomAD version V2.1.1). The variant was heterozygous in the sisters and the mother, not in the father. The second variant is a missense heterozygote G > C (c.G7400C) variant of uncertain significance in 47 exon of Calcium Voltage-Gated Channel Subunit Alpha 1 (CACNA1A) gene, resulting in a change from arginine to proline (p.R2467P). The nonsense variant has a frequency of 0.00002368 in The Genome Aggregation Database (gnomAD version V2.1.1). The variant was heterozygous in the sisters and the father, not in the mother. Algorithm developed to predict the effect of missense changes on protein structure and function result in the following: PolyPhen-2: “Damaging” (0.981). The amino acid residue arginine is found in multiple mammalian species, suggesting that this missense negatively affects the function of the protein (Supplementary Figure 2).

**Figure 3:**
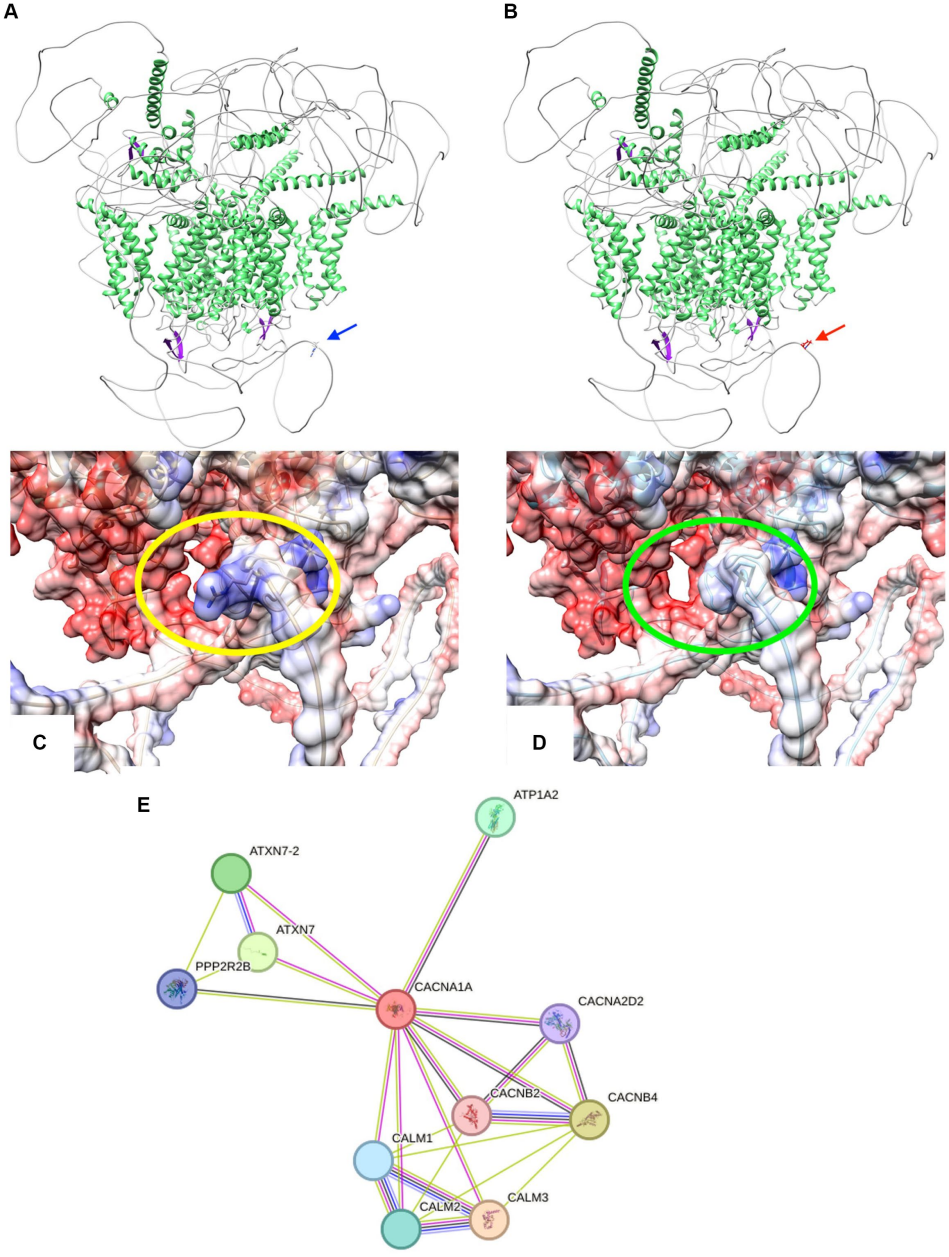
**(A,B)** Cartoon representations illustrate the protein structures of both CACNA1A wild-type (wt) and the R2467P mutant. The structures are color-coded by secondary elements: white represents loops, green indicates helices, and purple denotes sheets. On the left (blue arrow) is the wt structure, featuring the polar residue arginine. On the right (red arrow) is the R2467P mutant, characterized by the non-polar residue proline. **(C,D)** Surface representations depict the protein structures of both CACNA1A wild-type (wt) and the R2467P mutant. The structures are color-coded based on electrostatic values: red represents negative values (−10 min), white corresponds to 0, and blue signifies positive values (10 max). On the left (within the yellow oval) is the wt structure, featuring the polar residue arginine. On the right (within the green oval) is the R2467P mutant, characterized by the non-polar residue proline. Note the discernible modification in the electrostatic surface of this Intrinsically Disordered Region (IDR) caused by the mutation. **(E)** STRING network analysis of CACNA1A reveals a comprehensive interaction network involving proteins associated with its interaction. The connections are highlighted by co-expression (black), text mining (green), protein homology (purple), and experimentally determined interactions (pink).

Modeling the CACNA1A mutant isoform using Swiss Model and AlphaFold revealed an unchanged globular protein architecture with no discernible modifications in secondary motifs like beta turns and alpha helices (20, 21). Analysis showed the R2467P mutation alters the final loop, forming an intrinsically disordered region (IDR). This mutation changes residue 2,467 from a polar amino acid (arginine) to a non-polar proline with an amide lateral chain, impacting electron interaction, as seen in Figures 3A–E. Proline’s association with alpha helix formation suggests its role in maintaining IDR folding (22–24). Disorder in this region plays a crucial role in communication, scaffolding, and self-activation/inhibition (25–28). Disruption in CACNA1A’s communication within the extensive calcium equilibrium network may lead to neural electrophysiological perturbations, contributing to conductivity-related pathologies like seizures and epilepsy (29).

Based on the criteria outlined in the joint consensus of the ACMG (American College of Medical Genetics) and AMP (American Association of Molecular Pathology) for classifying pathogenic variants, we propose reclassifying this variant as a pathogenic Variant. It aligns with characteristics demonstrating supporting pathogenicity (PS1) as CACNA1A is associated with autosomal dominant Developmental and epileptic encephalopathy (OMIM: 617106), Episodic ataxia (OMIM: 108500), Migraine, familial hemiplegic with progressive cerebellar ataxia (OMIM: 141500), Spinocerebellar ataxia (OMIM: 183086) and Lennox–Gastaut syndrome (OMIM: 615369); (PS2) as the mother has one of the variants and the father has the other variant; its low frequency in control databases (PS4), and moderate evidence of pathogenicity (PM4) based on the conservation of the nucleotide.

## Discussion

Common neurological disorders of this age like generalized idiopathic seizures including juvenile myoclonic epilepsy and tonic–clonic seizures on awakening lack of cognitive and psychiatric alterations.

Both previous healthy adolescent patients presented with a progressive neurological disorder characterized by refractory epilepsy, cognitive decline, and ataxia, raising concerns beyond the common neurological disorder of this age, like generalized idiopathic seizures including juvenile myoclonic epilepsy and tonic–clonic seizures on awakening lack of cognitive and psychiatric alterations. Consequently, our investigation delved into mitochondrial encephalopathy, autoimmune encephalitis, and POLG-related disorders, with detailed characteristics outlined in Table 1. Mitochondrial encephalopathy, lactic acidosis, and stroke-like episodes syndrome (MELAS), a rare disorder commencing in childhood, primarily affecting the nervous system and muscles, manifest seizures as the predominant early symptoms, accompanied by stroke-like episodes featuring hemiparesis, altered consciousness, vision and hearing loss, loss of motor skills, and intellectual disability (30). Myoclonus Epilepsy associated with Ragged-red fibers (MERRF syndrome) is marked by myoclonic seizures, myopathy, ataxia, and dementia (30). Autoimmune encephalitis encompasses conditions where the immune system erroneously attacks healthy brain cells, causing brain inflammation with neurologic and/or psychiatric symptoms characterizing this condition (31). Finally, POLG-related disorders encompass various syndromes linked to a mutation in the nuclear POLG gene, representing the most prevalent single-gene cause of mitochondrial disease (32). Descriptions of six conditions falling under POLG-related disorders range from severe encephalopathy to ophthalmologic symptoms (32). This comprehensive exploration provides a foundation for understanding the unique neurologic and psychiatric features observed in our adolescent patients.

**Table 1:** Differential diagnosis comparison.

Disease	Mitochondrial encephalopathy, lactic acidosis, and stroke-like episodes (MELAS syndrome)	Myoclonic epilepsy and ragged red fibers	Leigh disease	Unverricht-lundborg disease	Lafora disease	Autoimmune encephalitis	Alpers-huttenlocher syndrome	Childhood myocerebrohepatopathy spectrum	Myoclonic epilepsy myopathy sensory ataxia	Ataxia neuropathy spectrum
Cause	Mutation in mitochondrial DNA – mutation in mtDNA gene MT-TL1 (80% of cases)	Mutation in mitochrondrial DNA – mutation in mtDNA lysine tRNA gene (MT-TK)	Mutations in mitochondrial DNA or by deficiencies of an enzyme called pyruvate dehydrogenase. The most common mtDNA change in Leigh syndrome keeps the MT-ATP6 gene from making ATP.	Mutations in the CSTB gene / autosomal recessive pattern	Mutation in either the EPM2A gene or the NHLRC1 gene	In many cases, the cause of autoimmune encephalitis is unknown.	Mutation in the POLG gene	Mutation in the POLG gene	Mutation in the POLG gene	Mutation in the POLG gene, or rarely mutation in the TWNK gene
Inheritance	Mitochondrial	Mitochondrial	Mitochondrial	Autosomal recessive pattern	Autsomal recessive pattern		Autosomal recessive pattern	Autosomal recessive pattern	Autosomal recessive pattern	Autosomal recessive pattern (POLG gene). Autosomal dominant pattern (TWNK gene)
Signs & symptoms	Symptoms usually begining between 2 and 15 years	Onset of MERRF is usually in childhood.	Early-onset (infantile): The most common form of Leigh syndrome appears before age 2. Providers also call it classical Leigh syndrome or infantile necrotizing encephalopathy. The condition affects boys and girls equally.	Affected individuals usually begin showing signs and symptoms of the disorder between the ages of 6 and 15.	The condition most commonly begins with epileptic seizures in late childhood or adolescence. Other signs and symptoms include difficulty walking, muscle spasms (myoclonus) and dementia. Affected people also experience rapid cognitive deterioration that begins around the same time as the seizures.	Neurologic symptoms may include impaired memory and cognition, abnormal movements, seizures, and/or problems with balance, speech, or vision. Psychiatric symptoms may include psychosis, aggression, inappropriate sexual behaviors, panic attacks, compulsive behaviors, euphoria or fear.	People with this condition usually have three characteristic features: recurrent seizures that do not improve with treatment (intractable epilepsy), loss of mental and movement abilities (psychomotor regression), and liver disease.	Typically becomes apparent in children from a few months to 3 years old. People with this condition usually have problems with their muscles (myo-), brain (cerebro-), and liver (hepato-).	Signs and symptoms of MEMSA typically appear during young adulthood.	(Neuropathy). The neuropathy can be classified as sensory, motor, or a combination of the two (mixed). Sensory neuropathy causes numbness, tingling, or pain in the arms and legs, and motor neuropathy refers to disturbance in the nerves used for muscle movement.
	Recurrence of stroke-like episones	Predominant progressive myoclonic epilepsy, a characteristic feature that separates MERRF from other mitochondrial diseases.	Late-onset (adult-onset): Symptoms appear after age 2 and may not occur until adolescence or early adulthood. Adult-onset Leigh syndrome is rare. The condition affects more males than females. The disease progresses slower than the infantile type. Symptoms may include: dementia, movemente and balance problems, dysartrhia, dystonia, muscle spasms, partial paralysis peripheral neuropathy, seizures	People with this disorder experience episodes of involuntary muscle jerking or twitching (myoclonus) that increase in frequency and severity over time. Episodes of myoclonus may be brought on by physical exertion, stress, light, or other stimuli		Some patients have antibodies in their blood or cerebrospinal fluid (CSF) which are known to be associated with encephalitis, while others test negative for antibodies but have characteristic symptoms.	Typically becomes apparent in children between ages 2 and 4.	Muscle weakness (myopathy), developmental delay or a deterioration of intellectual function, and liver disease.	The first symptom of MEMSA is usually cerebellar ataxia, which refers to problems with coordination and balance due to defects in the part of the brain that is involved in coordinating movement (cerebellum). Recurrent seizures (epilepsy) usually develop later, often in combination with uncontrollable muscle jerks (myoclonus)	Most people with ataxia neuropathy spectrum also have severe brain dysfunction (encephalopathy) and seizures
	Short stature and hearing loss may be present and fatigue and difficulty tolerating exercise	It is often photosensitive and aggravated by action and stimuli. Most of these patients also experience other types of seizures in addition to myoclonus. The seizures may be of generalized tonic-clonic, atonic or absence types		May develop problems with balance and coordination (ataxia), involuntary rhythmic shaking called intention tremor because it worsens during movement, difficulty speaking (dysarthria), depression, and a slow, mild decline in intellectual functioning.			Problems with coordination and balance (ataxia) and disturbances in nerve function (neuropathy).		Affected individuals may have severe brain dysfunction (encephalopathy) or muscle weakness (myopathy).	
	The most common early symptom are seizures, recurrent headaches, loss of appetite and recurrent vomiting. Stroke-like episodes with temporary muscle weakness on one side of the body, which can lead to altered consciousness, vision and hearing loss, loss of motor skills and intellectual disability	Commonly develop cerebellar ataxia, sensorineural deafness, short stature, cutaneous lipomas, and a clinical myopathy. cognitive decline and dementia also occur but late in the disease.								

Our WES analysis revealed a mutation in the AARS gene, encoding alanyl-tRNA synthetase critical for attaching alanine to its cognate tRNA in protein translation’s initial step (33). As a member of the highly conserved class II tRNA synthases family, alanyl-tRNA synthetase interprets the RNA code, attaching specific amino acids to tRNAs with cognate trinucleotide anticodons (34). Enzymes comprise a catalytic domain interacting with the tRNA’s amino acid acceptor-Tpsi C helix and a second domain interacting with the rest of the tRNA structure (34). Disease-causing mutations often affect the aminoacylation and editing domains (33). Four phenotypes linked to AARS gene mutations include Hereditary Diffuse Leukoencephalopathy with Spheroids (autosomal dominant), characterized by neurodegeneration and psychiatric symptoms (33–36); axonal Charcot–Marie–Tooth disease type 2 (autosomal dominant), a common inherited neuropathy (37–40); Developmental and Epileptic Encephalopathy, an autosomal recessive neurologic disorder (41, 42); and Nonphotosensitive Trichothiodystrophy, an autosomal recessive disorder with characteristic features (43, 44). Despite sharing the gene mutation, our patients do not fit these phenotypes. These healthy females exhibited typical motor and neurological development until age 15, displaying progressive symptoms from seizures to motor and cognitive alterations. Patient B presented additional psychiatric symptoms. In the ClinVar database, we identified 27 nonsense variants of the AARS gene: 16 classified as pathogenic, three as likely pathogenic, nine as variants of uncertain significance, and one with conflicting classifications. These nonsense variants have been associated with conditions such as Charcot–Marie–Tooth disease type 2, Developmental and epileptic encephalopathy type 29, axonal type 2N Charcot–Marie–Tooth disease, and various inborn genetic diseases. The variant most like ours was categorized as pathogenic for Charcot–Marie–Tooth disease type 2, resulting in a p.Y690* mutation (NC_000016.10: 70258139: A > C).

Our patients harbor a second mutation in the CACNA1A gene, responsible for encoding the transmembrane pore-forming subunit of the P/Q-type or CaV2.1 voltage-gated calcium channel (45). These voltage-dependent Ca (2+) channels play a crucial role in facilitating Ca (2+) ion entry into excitable cells and participate in various Ca (2+)-dependent processes, including muscle contraction, hormone or neurotransmitter release, and gene expression (46). Mutations in this gene were associated with cerebellar ataxia, hemiplegic migraine and epileptic encephalopathy, demonstrating a broad phenotypic spectrum with autosomal dominant inheritance (13–17). The pathogenic variant in CACNA1A (c.C835T, p.R279C) has been documented to result in diverse manifestations among carriers, ranging from severe epileptic encephalopathy to episodic ataxia and cerebellar ataxia with intellectual deficiency, highlighting significant incomplete penetrance (14). Functional studies conducted in mouse thalamic relay neurons shed light on the impact of different CACNA1A variants, underscoring the pivotal role of calcium channels in synaptic dysfunction and clinical manifestations (47).

Both of our patients present with a mutation in the AARS gene, which is also presented in their mom, but they are the first of their mother’s family side with symptoms suggesting incomplete penetrance and an autosomal dominant inheritance. Additionally, both patients present with a mutation in the CACNA1A gene, which is also present in their father. The latter presented with migraines during his teenage years, and four other family members of their father’s family side have reported seizures. Comprehensive whole exome sequencing was performed, thoroughly examining all genes associated with the clinical symptoms and additional genes as specified previously. This led us to identify AARS and CACNA1A as the only genes likely to be implicated in the etiology of the condition. We speculate that having these two mutations could explain the fast progression of symptoms and deterioration.

Two crucial lessons emerge from these cases. Firstly, the significance and efficacy of genetic testing for diverse phenotypes become evident. In our patients, the implementation of Whole Exome Sequencing (WES) proved essential for achieving a definitive diagnosis. Unfortunately, in Latin American countries like Ecuador, access to genetic specialists and genetic testing is limited, underscoring the necessity for forming collaborative partnerships with specialists from other countries (48). This collaborative approach aims to promote universal, equitable, and accessible healthcare for all individuals. Secondly, the family history of our patients underscores the importance of meticulous family history documentation and the utilization of pedigrees. These tools aid in identifying other family members exhibiting related symptoms, deciphering the pattern of transmission, and identifying potential health problems that an individual may be at increased risk for in the future (38). Thus, emphasizing the significance of comprehensive family history assessment contributes to a more accurate understanding of genetic conditions and facilitates proactive health management.

## Conclusion

Our study unravels a complex interplay of genetic factors contributing to a novel phenotype in two adolescent patients with a dual mutation in the AARS and CACNA1A genes. The distinct progression of symptoms from seizures to motor and cognitive alterations, including psychiatric symptoms in Patient B, challenges the conventional understanding of AARS-related phenotypes. The CACNA1A mutation further adds to the complexity, showcasing a broad phenotypic spectrum with significant incomplete penetrance. The familial inheritance of these mutations correlates with an accelerated symptom progression, emphasizing their potential role in the clinical manifestation. We underscore the essential role of genetic testing, especially Whole Exome Sequencing, in diagnosing diverse phenotypes, highlighting the need for international collaborations to improve healthcare accessibility, and emphasizes the significance of detailed family history documentation for a comprehensive and collaborative approach to genetic research and healthcare, ensuring universal access and proactive management for individuals and families with complex genetic conditions.

## Data availability statement

The datasets presented in this article are not readily available because of ethical and privacy restrictions. Requests to access the datasets should be directed to the corresponding author.

## Ethics statement

The studies involving humans were approved by CEISH Universidad San Francisco de Quito. The studies were conducted in accordance with the local legislation and institutional requirements. Written informed consent for participation in this study was provided by the participants’ legal guardians/next of kin. Written informed consent was obtained from the minor(s)’ legal guardian/next of kin for the publication of any potentially identifiable images or data included in this article.

## Author contributions

VR: Conceptualization, Data curation, Formal analysis, Funding acquisition, Investigation, Methodology, Project administration, Resources, Software, Supervision, Validation, Visualization, Writing – original draft, Writing – review & editing. SS: Data curation, Investigation, Visualization, Writing – original draft, Writing – review & editing. BA-A: Formal analysis, Validation, Visualization, Writing – original draft, Writing – review & editing. DD: Conceptualization, Visualization, Writing – original draft, Writing – review & editing. KH: Data curation, Formal analysis, Funding acquisition, Methodology, Writing – original draft, Writing – review & editing.
