# Supplementary material for: AARS and CACNA1A mutations: diagnostic insights into a case report of uncommon epileptic encephalopathy phenotypes in two siblings
Source: Front Neurol. 2024 Apr 15;15:1376643. doi: 10.3389/fneur.2024.1376643 (PMC11059961; doi:10.3389/fneur.2024.1376643)

**Supplementary figures**

**Supplementary figure 1. CARE timeline**

**
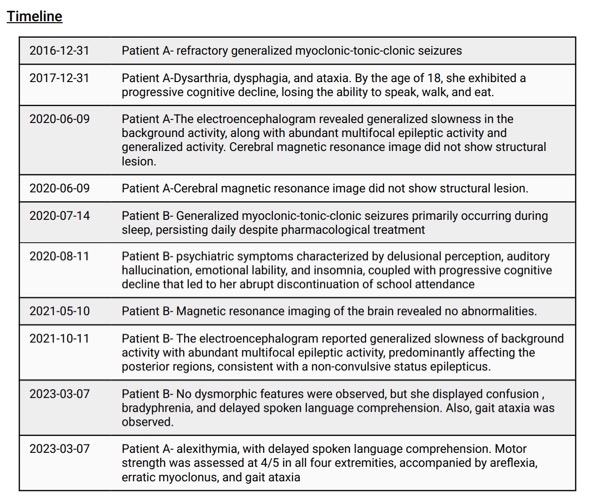
**

**Supplementary figure 2.** USCS database screenshot of the variant in CACNA1A gene. The variant is conserved in various animals including dog, elephant and zebrafish.


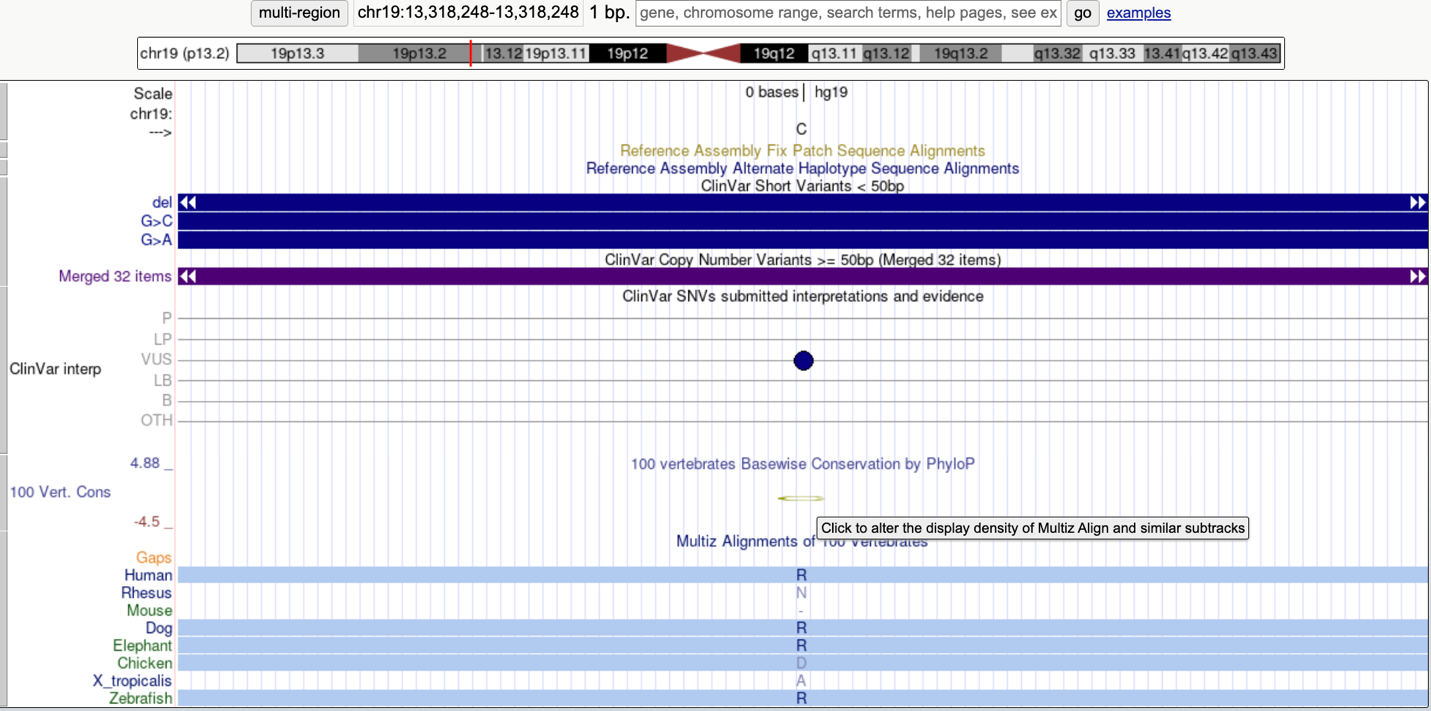

Supplement: Supplementary file 1 [file Data_Sheet_1.docx]
